# Decreasing household contribution to TB transmission with age: a retrospective geographic analysis of young people in a South African township

**DOI:** 10.1186/1471-2334-14-221

**Published:** 2014-04-23

**Authors:** Keren Middelkoop, Linda-Gail Bekker, Carl Morrow, Namee Lee, Robin Wood

**Affiliations:** 1 Desmond Tutu HIV Centre, Institute of Infectious Disease & Molecular Medicine, University of Cape Town, Cape Town, South Africa; 2 Department of Medicine, University of Cape Town, Cape Town, South Africa; 3 Human Biology Department and Cell & Systems Biology Department, University of Toronto, Toronto, Canada

**Keywords:** Tuberculosis, Infection, Transmission, Adolescents

## Abstract

**Background:**

Tuberculosis (TB) transmission rates are exceptionally high in endemic TB settings. Adolescence represents a period of increasing TB infection and disease but little is known as to where adolescents acquire TB infection. We explored the relationship between residential exposure to adult TB cases and infection in children and adolescents in a South African community with high burdens of TB and HIV.

**Methods:**

TB infection data were obtained from community, school-based tuberculin skin test (TST) surveys performed in 2006, 2007 and 2009. A subset of 2007 participants received a repeat TST in 2009, among which incident TB infections were identified. Using residential address, all adult TB cases notified by the community clinic between 1996 and 2009 were cross-referenced with childhood and adolescent TST results. Demographic and clinic data including HIV status were abstracted for TB cases. Multivariate logistic regression models examined the association of adult TB exposure with childhood and adolescent prevalent and incident TB infection.

**Results:**

Of 1,100 children and adolescents included in the prevalent TB infection analysis, 480 (44%) were TST positive and 651 (59%) were exposed to an adult TB case on their residential plot. Prevalent TB infection in children aged 5–9 and 10–14 years was positively associated with residential exposure to an adult TB case (odds ratio [OR]:2.0; 95% confidence interval [CI]: 1.1-3.6 and OR:1.5; 95% CI: 1.0-2.3 respectively), but no association was found in adolescents ≥15 years (OR:1.4; 95% CI: 0.9-2.0). HIV status of adult TB cases was not associated with TB infection (p = 0.62). Of 67 previously TST negative children, 16 (24%) converted to a positive TST in 2009. These incident infections were not associated with residential exposure to an adult TB case (OR: 1.9; 95% CI: 0.5-7.3).

**Conclusions:**

TB infection among young children was strongly associated with residential exposure to an adult TB case, but prevalent and incident TB infection in adolescents was not associated with residential exposure. The HIV-status of adult TB cases was not a risk factor for transmission. The high rates of TB infection and disease among adolescents underscore the importance of identifying where infection occurs in this age group.

## Background

Despite recent improvements in global tuberculosis (TB) trends, this disease remains responsible for significant morbidity and mortality in many African countries [1]. In order to achieve TB control, it is necessary for an increasing proportion of the population to remain free of infection [2]. Reaching this goal in Africa will require the development and implementation of strategies adjunctive to Directly Observed Treatment Short-course (DOTS) that reduce TB transmission. However, while it is recognized that *Mycobacterium tuberculosis* (*Mtb*) transmission rates are exceptionally high [3], there are few data on the drivers and determinants of *Mtb* transmission in high HIV burdened communities.

We have previously suggested that age-specific interventions may be required for TB control, and this is particularly applicable in the context of *Mtb* transmission. It is well documented that *Mtb* transmission to children occurs predominantly in households [4-6], and therefore household-based interventions may be most appropriate for young children [7]. In contrast, there are few data exploring where adolescents acquire TB infection. Yet adolescence represents a period of increasing risk of both TB disease [8] and infection. Studies from sub-Saharan Africa report high prevalence [3] and incidence [9] of TB infection in this age group, and adolescents are increasingly recognized as a key target group for future TB prevention initiatives [10].

We aimed to determine the importance of household TB contacts in TB transmission to children and adolescents in a community with high burdens of TB and HIV disease.

## Methods

### Study community

The study community has been described in detail elsewhere, but in brief, it is a recently urbanised, overcrowded, geographically well-demarcated peri-urban township. The population is of low socio-economic status and the housing is largely comprised of informal dwellings. The HIV prevalence measured in 2005 and 2010 was 23% among adults [11,12] and was 5% among secondary school-going adolescents in 2009 [3]. TB notification was mandatory throughout the study period, and TB rates are exceptionally high in this community, ±2,000/100,000 since 2006 [13]. There is a mean annual risk of TB infection of 4.1%, and we have previously shown that the annual incidence of TB infection increased with age among children and adolescents [14].

The community consists of a formal sector with demarcated, individually numbered plots with electricity, water and plumbing services, and an informal sector of shacks sharing communal services. In this community residential erfs are plots of land approximately 180 m^2^ which may contain from 1 to 22 houses (mostly informal housing but also a mix of brick houses and shack dwellings), with a mean of 10 residents per plot. Plots are frequently shared by extended families, representing a broadened but intimate social environment. This analysis was restricted to residents in the formal sector (78% of residents in 2008 community census - unpublished).

Childhood and adolescent TB infection data were obtained from school-based tuberculin skin test (TST) surveys performed in the community primary school in 2006 and 2007 [14] and in the secondary school in 2009 [3]. HIV testing was performed in the secondary school survey but not in the primary school, where most children were presumed to be HIV-uninfected [14]. Secondary school participants who tested HIV-infected were excluded from analysis and those who declined HIV testing were considered to be HIV-uninfected for the purposes of the TB analysis. A subset of students who had participated in the 2007 survey also participated in the 2009 survey, resulting in repeat tuberculin testing of these participants. Therefore TB infection was defined in two groups: prevalent and incident infection. Prevalent TB infection was defined as a TST positive result (an induration ≥10 mm in response to 2 units of purified protein derivative administered intradermally [14]) identified during first participation in a TST survey. Among the children who participated in two surveys, those who tested TST negative in the first survey were then included in the incident TB infection analysis. In keeping with existing literature, we defined an incident TB infection as a change from a negative result (<10 mm) on the first TST to a positive result (≥10 mm) on the second test, with an absolute reaction size increase of at least 6 mm [15-17]. Participants were divided into three age categories: 5–9, 10–14 and ≥15 years of age. Children were defined as participants <15 years of age and adolescents were those school-going participants 15 to 22 years old. These categories were based on social mixing data from the community, which showed an increase in mean number of indoor contacts from 5–9 to 10–14 year olds, but a marked increase among adolescents ≥15 years of age [18].

All adult TB cases notified by the community TB clinic between 1996 and 2009 were cross-referenced with childhood and adolescent TST results from the school surveys using residential address. For each child the search was restricted to the period of the child’s lifetime. Adult TB cases were defined as patients ≥15 years of age, as per WHO definitions [1]. Demographic and clinical data including age, gender, TB clinical diagnosis and HIV status were collected from the TB register and clinic records. Adult TB cases were defined into three mutually exclusive categories: smear-positive pulmonary TB (PTB), smear negative PTB and extra-pulmonary TB (EPTB) cases. Patients with both PTB and EPTB were classified in the appropriate PTB category.

These studies were approved by the University of Cape Town’s Human Research Ethics Committee. Parental consent and participant assent was obtained from all participating children, and adult TB case information was obtained from TB notification data.

### Data analysis

Data were analyzed using STATA 11.0 (StataCorp, College Station, Texas). Bivariate analyses employed Wilcoxon rank sum and chi-squared tests, as appropriate. Multivariate logistic regression models were developed to examine the association of adult TB exposure on the residential plot with childhood and adolescent prevalent and incident TB infection. Adult TB cases were assessed as binary exposures (ie exposure to one or more TB case of a particular category). Models were adjusted for clustering effect on plots. All statistical tests were 2-sided at alpha = 0.05. The ArcMap 10 (Esri™) Geographic Information System was used to visualize the spatial distribution of adolescent TB infection with adult cases in the community.

## Results

### Childhood and adolescent TB infection cohort

**Figure 1:**
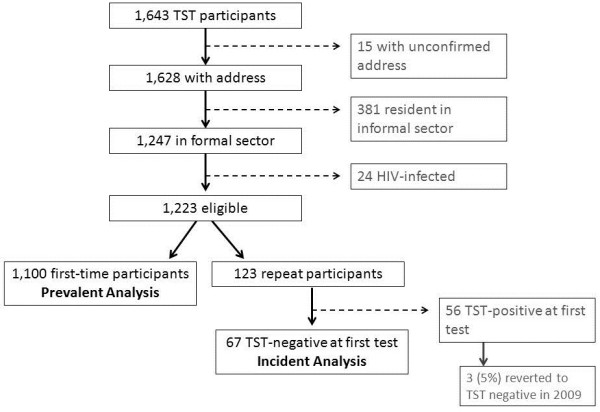
Consort diagram of children and adolescent TB infection cohort.

In total 1,643 children and adolescents completed tuberculin skin testing in the three surveys. Of these 15 (1%) did not have a confirmed address and 381 (23%) lived in the informal sector of the community and were therefore excluded from analysis (Figure 1). Those living in the formal sector did not differ from those living in the informal sector in terms of TB infection (p = 0.17), age (p = 0.10), gender (p = 0.74) and, among secondary school participants, HIV infection (p = 0.14). Of the secondary school children, 24 (5%) of the 477 adolescents in the formal sector who were tested were HIV-positive and were excluded from further analyses.

### Adult TB contact cases

Overall 2,011 adult cases were notified in the community from 1996 to 2009, of which 1,604 lived in the formal sector. Adult TB patients resident in the formal sector did not differ from those in the informal sector in terms of age (p = 0.49), gender (p = 0.95), new or retreatment TB (p = 0.35), TB treatment outcome (p = 0.20), multi-drug resistant (MDR)-TB (p = 0.43), or HIV status (p = 0.42). Smear-negative PTB was under-represented in the formal sector of the community (15% vs 20% in informal sector; p = 0.03), but smear-positive PTB and EPTB did not differ between the two sectors (p = 0.73).

In total 766 adult cases were contact cases, sharing a residential plot with one or more TST participants living in the formal sector of the community. The median age of these adults was 33 (interquartile range [IQR]: 26–40) and 46% were female. Of the 575 adults who had tested for HIV, 381 (66%) were positive. In total, 559 (73%) of the cases were new cases (first TB episode). Furthermore 441 (58%) had confirmed smear-positive PTB, 281 (37%) had smear-negative PTB, and 44 (6%) had EPTB. MDR-TB was confirmed in 13 (2%) cases.

### Prevalent TB infection

Of the 1,223 eligible TST participants, 1,100 (90%) were first-time participants, and were included in the prevalent TB infection analyses (Figure 1). The median age of these participants was 14 (IQR: 10–17) and 50% were female. Of these children and adolescents, 480 (44%) were TST positive at their first TST. The distribution of TST positivity by age categories is shown in Table 1.

**Table 1:** TB infection prevalence, exposure to adult TB case on residential plot and association between TB infection and residential TB case exposure by age categories

**Age category**		**TST positive**		**Exposed to TB on plot**		**Odds of TB infection in those exposed to adult TB on plot**		**Odds of TB infection in those exposed to smear positive PTB on plot**	
	**n**	**n (%)**		**n (%)**		OR* (95% CI)		OR* (95% CI)	
**5-9**	258	74	29%	144	56%	2.0	(1.1-3.6)	2.0	(1.1-3.5)
**10-14**	370	151	41%	209	46%	1.5	(1.0-2.3)	2.4	(1.5-3.6)
**≥15**	472	255	54%	298	63%	1.4	(0.9-2.0)	1.4	(0.9-2.0)
**Total**	**1,100**	**480**	**44%**	**651**	**59%**	**1.6**	**(1.2-2.0)**	**1.9**	**(1.4-2.4)**

*Odds ratios adjusted for gender of the child/adolescent.

### Prevalent TB infection and residential exposure

Of the 1,100 children and adolescents included in the prevalent TB analysis, 651 (59%) were exposed to one or more adult TB case on their residential plot (Table 1). Children were exposed to a median of one adult case on their plot (IQR: 0–1), but the maximum number of residential contacts was nine. Overall 492 (45%) were exposed to smear-positive PTB, 252 (23%) were exposed to smear-negative PTB, and 196 (18%) to EPTB.

**Figure 2:**
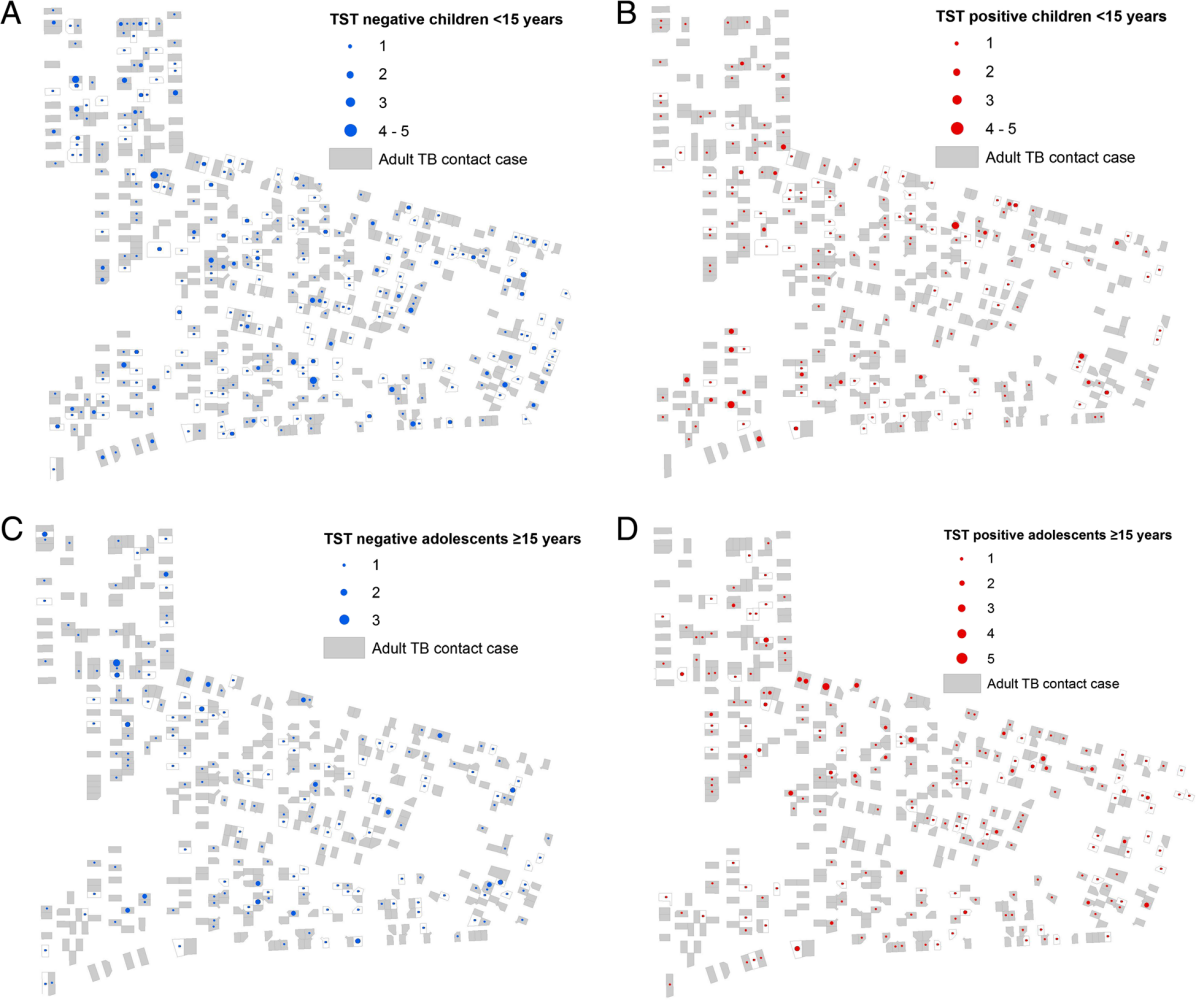
**Distribution of children TST negative (a) and TST positive (b) and adolescent TST negative (c) and TST positive (d) results and of adult TB cases from 1997 to 2009 in the community.** Overall, 52% of TST negative children were exposed to an adult TB case on their residential plot compared to 64% of TST positive children (p = 0.003), and 59% of TST negative adolescents were exposed to an adult TB case on their residential plot compared to 66% of TST positive adolescents (p = 0.13).

In multivariate analysis adjusted for the gender of the child, prevalent TB infection (as defined by TST positivity) in children aged 5 to 9 and 10 to 14 years of age was positively associated with exposure to any adult TB case on their residential plot (odds ratio [OR]: 2.0; 95% confidence interval [CI]: 1.1-3.6 and OR:1.5; 95% CI: 1.0-2.3 respectively). Specifically, prevalent TB infection among these children was associated with exposure to smear-positive PTB cases (Table 1). No statistical association between TB infection and exposure to adult TB cases, including smear-positive cases, was found in adolescents 15 years and older (OR for any adult TB exposure: 1.4; 95% CI: 0.9-2.0). Figure 2 shows the distribution of adult TB cases and prevalent childhood and adolescent TB infection status in the community. In all age categories there was no statistical association between childhood/adolescent TB infection and exposure to adult smear-negative PTB or EPTB cases on the residential plot (data not shown).

In multivariate models that adjusted for the HIV status of adult TB cases, prevalent TB infection remained significantly associated with exposure to any adult TB case and smear-positive adult TB cases, but HIV status of the TB case was not associated with TB infection (p = 0.62 and 0.65 respectively). HIV-positive contact cases were less likely to be smear-positive (p < 0.001).

### Incident TB infection

Of the 123 TST participants who tested in 2007 and had a repeat TST in 2009, 67 were TST negative in 2007 and therefore comprised the incident TB infection analysis (Figure 1). The median age of this group was 15 (IQR: 15–16) and 51% were female. Of these adolescents 16 (24%; 95% CI: 14-36%) converted to a positive TST result in 2009. This is an annual incidence of 12%.

### Incident TB infection and residential exposure

Of the 67 adolescents included in the incident TB analysis, 14 (21%) were exposed to one or more adult TB case on their residential plot and 6 (9%) were exposed to smear-positive PTB. In multivariate analysis adjusted for the gender of the adolescent, incident TB infection was not statistically associated with exposure to either any adult TB case (OR:1.9; 95% CI: 0.5-7.3) or smear-positive PTB cases (OR:1.5; 95% CI: 0.3-8.4) on their residential plot.

## Discussion

This is one of the first studies to report on the risk of household TB transmission to adolescents resident in a high TB burdened community. In keeping with a previous report [19], TB infection among young children remained strongly associated with exposure to an adult TB case on their residential plot. However, the main finding of this study was that while adolescents are exposed to high numbers of adult TB cases on their residential plots, both prevalent and incident TB infection in adolescents was not strongly associated with this exposure. Furthermore, the HIV-status of adult TB cases was not associated with either an increased or decreased risk of transmission to children, even when adjusted for smear-positive status.

We have previously reported that TB infection in younger children was strongly associated with presence of an adult case on the plot [19] and that finding persisted in this study. In particular, childhood infection was strongly associated with exposure to smear-positive adult TB cases on residential plot. However, both prevalent and incident TB infection among adolescents (15 to 22 years) was not significantly associated with the presence of an adult case on the plot. This finding is in keeping with a social mixing study from this community which reported that younger children have more indoor contact hours on their residential plots compared to adolescents [18]. Local and European reports show that the number of non-family social contacts peak in adolescence [18,20] and adolescence represents an age of increasing social interaction outside of the home that may more closely resemble that of adults rather than younger children in the community. This is consistent with a molecular epidemiological study that confirmed that young children are significantly more likely to acquire TB infection from a household contact compared to adolescents [21], as well as with molecular epidemiological data from this community that showed that adult to adult transmission on residential plots is low (approximately 10% - unpublished data).

The annual incidence of TB infection among adolescents who had previously tested TST negative was 12% – higher than the annual infection rate of up to 7% previously reported for this community [3]. However, cross-sectional surveys are unable to measure repeated exposures and infection in individuals. Furthermore, the rate of TST reversion in this community is as high as 5%, resulting in an underestimate of incident infections modeled on TST cross-sectional data. The dynamic process illustrated by these two concepts may, in part, explain the discrepancy between these two measures of TB infection incidence, and it is likely that cross-sectional TST studies underestimate the substantial transmission pressure experienced in these high burdened communities.

There are conflicting reports on the infectiousness of HIV-positive TB patients [22,23] and therefore another important study finding was that HIV status of adult TB contact cases was not an independent risk factor for childhood TB infection. This is in keeping with the previous finding in this community that despite increasing rates of adult HIV-associated TB, childhood TB notification rates have remained relatively constant over the past decade [14].

There are some limitations to this study. The analysis was restricted to the formally serviced sector of the community. As shack identifiers in the informal sector have not remained constant over the study period we could not define the residential area of interaction between adults and children living in these areas. However, no significant differences were noted in either the TST participants or adult TB cases across the formal and informal sectors, limiting any potential selection bias. In addition, the formal sector is comprised largely of informal dwellings and furthermore, it is unlikely that there are significant differences in social dynamics across the two community areas.

A further limitation is that we were unable to confirm the stability of residential status over the study period. In particular it is possible that older adolescents were not living on the same plot throughout the study period, and this may partially explain the weaker association between adult contact cases and prevalent adolescent TB infection. Furthermore, adult contact cases were censored for the children’s lifetime. A higher proportion of adolescents were therefore exposed to adult TB on their plots compared to younger children, due to their increased time in the community. This time bias may be a confounder in the prevalent TB data analysis; however, one would anticipate it may artificially increase the association between adolescent TB infection and exposure on plots. In contrast, we found no association, and this finding is strengthened by the lack of association between adolescent incident TB infection that restricted analysis to residential TB exposure occurring in the two years subsequent to a negative TB infection test result.

## Conclusion

In conclusion, this study reported that in high TB and HIV burdened communities adolescent TB infection was not significantly associated with exposure to adult TB cases on their residential plots, suggesting increasing importance of non-residential TB exposure. An extremely high annual incidence rate of TB infection was noted among adolescents and this finding combined with the well-known risk of TB disease in young adolescents and adults [8], underscores the importance of identifying high-risk locations for infection in this age group.

## Competing interest

The authors declare that they have no competing interests.

## Authors’ contributions

KM: Literature search, data collection, data analysis and interpretation, GIS figures, manuscript first draft. LGB: Study design, data interpretation, manuscript review. CM: Data management, assisted with GIS figures, manuscript review. NL: Literature search, data analysis, manuscript review. RW: Study design, data interpretation, manuscript review. All authors read and approved the final manuscript.

## Pre-publication history

The pre-publication history for this paper can be accessed here:

http://www.biomedcentral.com/1471-2334/14/221/prepub
